# The ECG–echocardiography interval in EchoNext-Mini validation

**DOI:** 10.1093/ehjdh/ztag175

**Published:** 2026-10-30

**Authors:** Hasan Burak Isleyen, Sercan Bulut

**Affiliations:** Department of Cardiology, Istanbul Nisantasi University Faculty of Medicine, Istanbul 34398, Türkiye; Department of Cardiology, Bezmialem Vakif University Faculty of Medicine Hospital, Istanbul, Türkiye; Cardiology Clinic, Bağcılar Training and Research Hospital, Istanbul, Türkiye

Otabor and colleagues externally validated EchoNext-Mini in 45 878 patients from MIMIC-IV.^1^ The authors linked each electrocardiogram (ECG) to a subsequent eligible echocardiogram within one year. Table 1 reports a median interval of 2 days (IQR 1–15 days), indicating that approximately three quarters of the cohort underwent echocardiography within 15 days. The authors also appropriately describe the cohort as clinically selected rather than an unselected screening population. These points narrow, but do not eliminate, the interpretive question posed by the one-year linkage window.

An ECG obtained close to echocardiography can be evaluated as a screen for structural disease present at the time of testing. When echocardiography occurs many months later, the label may reflect interval progression, treatment, or a new clinical event. The remaining selection issue is therefore not whether referral occurred, but how results from patients already referred for echocardiography should inform intended use. Clinical interpretation should remain focused on similarly selected patients unless performance is established in an unselected screening population.

Supplementary Table S8 of Otabor et al.^1^ reports outcome prevalence, area under the receiver operating characteristic curve (AUROC), calibration-in-the-large (CIL), calibration slope, and Brier skill for the 30-, 90-, 180-, and 365-day linkage windows. Composite AUROC was 0.789, 0.791, 0.792, and 0.790, respectively; calibration slope remained 0.93–0.94 and CIL +0.023 to +0.028. These stable estimates are reassuring. However, the windows are nested rather than mutually exclusive and are presented without stratum denominators or confidence intervals. Consequently, patients with the longest ECG-to-echocardiography intervals are not shown separately, and the precision of comparisons between windows cannot be judged. Mutually exclusive interval strata with denominators and uncertainty, if readily available, would be informative; at minimum, the implications of the published linkage-window analyses merit explicit discussion.

Prevalence-based recalibration of component probabilities addresses one aspect of transportability.^1^ It cannot, by itself, determine whether a one-year subsequent echocardiographic label represents disease present when the ECG was recorded. The article does not claim otherwise. We therefore suggest clarifying the distinction between contemporaneous diagnostic or prioritization use at the ECG date and interpretation of disease documented later within the one-year window. This is a clarification of intended clinical use rather than a correction, and does not imply that the study evaluated incident disease.

## Lead author biography


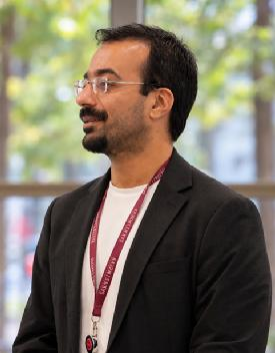


Hasan Burak Isleyen, MD *Assistant Professor of Cardiology* Asst. Prof. Hasan Burak Isleyen, MD, is an Assistant Professor of Cardiology at Istanbul Nisantasi University Faculty of Medicine and is also affiliated with the Department of Cardiology at Bezmialem Vakif University Faculty of Medicine Hospital, Istanbul, Türkiye. His academic interests include artificial intelligence and digital health in cardiology, electrocardiography, echocardiography, cardiovascular risk stratification, and clinically oriented cardiovascular research.

## Data Availability

No new data were generated or analysed.
